# The Toll-Like Receptor Agonist Imiquimod Is Active against Prions

**DOI:** 10.1371/journal.pone.0072112

**Published:** 2013-08-16

**Authors:** Nassima Oumata, Phu hai Nguyen, Vincent Beringue, Flavie Soubigou, Yanhong Pang, Nathalie Desban, Catherine Massacrier, Yannis Morel, Carine Paturel, Marie-Astrid Contesse, Serge Bouaziz, Suparna Sanyal, Hervé Galons, Marc Blondel, Cécile Voisset

**Affiliations:** 1 Laboratoire de Chimie Organique 2, INSERM U1022, Université Paris Descartes, Paris, France; 2 Institut National de la Sante et de la recherche Medicale UMR1078, Université de Bretagne Occidentale, Faculté de Médecine et des Sciences de la Santé ; Etablissement Français du Sang (EFS) Bretagne ; CHRU Brest, Hôpital Morvan, Laboratoire de Génétique Moléculaire, Brest, France; 3 Virologie Immunologie Moléculaires, UR892, Institut National de la Recherche Agronomique (INRA), Jouy-en-Josas, France; 4 Department of Cell and Molecular Biology, BMC, Uppsala University, Uppsala, Sweden; 5 Protein Phosphorylation & Disease Laboratory, CNRS UPS2682, Roscoff, France; 6 Innate Pharma, Marseille, France; 7 UMR 8015 CNRS, Laboratoire de Cristallographie et RMN Biologiques, Université Paris Descartes, Paris, France; University of Maryland School of Medicine, United States of America

## Abstract

Using a yeast-based assay, a previously unsuspected antiprion activity was found for imiquimod (IQ), a potent Toll-like receptor 7 (TLR7) agonist already used for clinical applications. The antiprion activity of IQ was first detected against yeast prions [*PSI* ^*+*^] and [URE3], and then against mammalian prion both *ex vivo* in a cell-based assay and *in vivo* in a transgenic mouse model for prion diseases. In order to facilitate structure-activity relationship studies, we conducted a new synthetic pathway which provides a more efficient means of producing new IQ chemical derivatives, the activity of which was tested against both yeast and mammalian prions. The comparable antiprion activity of IQ and its chemical derivatives in the above life forms further emphasizes the conservation of prion controlling mechanisms throughout evolution. Interestingly, this study also demonstrated that the antiprion activity of IQ and IQ-derived compounds is independent from their ability to stimulate TLRs. Furthermore, we found that IQ and its active chemical derivatives inhibit the protein folding activity of the ribosome (PFAR) *in vitro.*

## Introduction

Prions are infectious agents that have been affecting human and animal health for centuries. Prion diseases, also named transmissible spongiform encephalopathies (TSEs), are invariably fatal and no treatment is currently available. Today, the ability of the new variant of Creutzfeldt–Jakob disease to be transmitted by blood transfusion is well documented [1]. In face of potential new TSE outbreaks due to this or other secondary infection routes [2], our current therapeutics are limited. More efficient therapies are urgently needed.

As a result of the life span increase, age-related neurodegenerative diseases impose a considerable burden on our societies. Many such diseases are proteinopathies, defined as conditions in which accumulation of a protein in a misfolded form leads to neuronal degeneration. The misfolded proteins present in these neurodegenerative diseases as well as in related systemic (non-neuronal) diseases, assemble into highly ordered supramolecular assemblies called amyloids. Amyloids are partially resistant to proteolytic degradation and can template the further recruitment of protein or peptide monomers into the polymers. These templating abilities are reminiscent of those of prion diseases and suggest that propagation of neurodegenerative disorders may occur in a “prion-like” manner through mechanisms similar to those that underlie prion pathogenesis [3–8]. A better understanding of the mechanisms of prion propagation is therefore crucial for the development of therapeutic strategies to prevent the propagation of misfolded proteins in the brain and in other organs.

Our previous studies have detected compounds active against yeast and mammalian prions [9–12]. Briefly, compounds from chemical libraries have been tested for their ability to cure primarily [*PSI* ^+^] (primary screening) and then [URE3] (secondary screening) yeast prions. Compounds displaying an antiprion activity against both [*PSI* ^*+*^] and [URE3] yeast prions have been further tested for their ability to clear pathogenic mammalian prion protein PrP^Sc^ in a cell-based assay using chronically prion infected MovS6 cells [12,13]. Drugs that have a potent antiprion activity in both yeast- and *ex vivo* cell-based assays, and that display a limited or no toxicity *ex vivo*, have then been tested *in vivo* in a mouse model for prion-based diseases. These compounds have also been used to characterize their cellular targets [11]. The initial steps of the screening strategy are based on two yeast prions that are related neither in sequence nor function and have no link with PrP. Thus, isolated compounds active against yeast and mammalian prions should most probably target cellular prionisation mechanisms and not the prion proteins themselves. We have indeed isolated compounds able to inhibit prion propagation in both yeast and mammalian systems [9–12], indicating that at least part of prionisation mechanisms are preserved from yeast to mammals. Thus, the use of such a screening process as a complement to animal-based models is highly fruitful and has been successfully used for other pathologies [14]. This screening procedure has allowed the identification of two active drugs, 6-aminophenanthridine (6AP) and Guanabenz (GA), that have proven to be valuable tools to identify a novel cellular mechanism potentially involved in the prion replication cycle, i.e. the protein folding activity of the ribosome (PFAR, [11,13,15,16]).

Here, by testing a library of drugs already on the market, we found Imiquimod (IQ), a potent Toll-like receptor agonist, to be active against both yeast and mammalian prions. We also set up a new and efficient synthesis pathway of IQ to ease its production and to initiate the synthesis of its new derivatives so far difficult to produce. The antiprion activity of prepared IQ chemical derivatives as well as other TLR7/8 agonists such as gardiquimod was evaluated against yeast and mammalian prions. The potential of IQ chemical derivatives to stimulate TLR7/8 was also assessed. These observations revealed that antiprion activity of the tested compounds do not overlap with their agonist activity on TLR7/8 receptors. Furthermore, we found that IQ and its chemical derivatives, acting as antiprions, were specific inhibitors of the protein folding activity of the ribosome, in addition to the already known TLR7 agonist activity of IQ.

## Results and Discussion

### Imiquimod is active against yeast and mammalian prions

**Figure 1:**
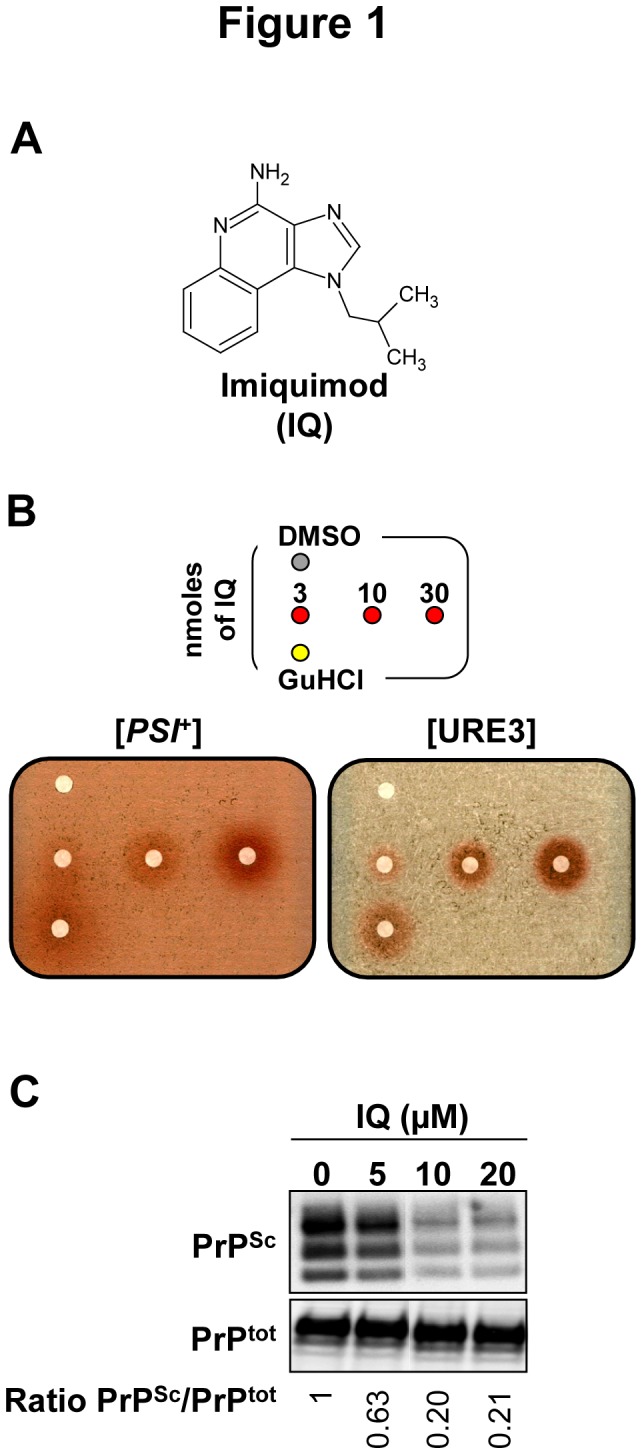
Imiquimod (IQ) is active against both yeast and mammalian prions. **A**. Molecular structure of IQ. **B**. [*PSI* ^+^] (left panel) and [URE3] (right panel) strains were spread on YPD medium supplemented with 200 and 800 µM GuHCl, respectively. Small filters were then placed on the agar surface and increasing amounts of IQ were applied to each filter, except for the top left filter where DMSO, the compounds vehicle was added (negative control) and for the bottom left filter where GuHCl was added (positive control). The apparition of a halo of red colonies around a filter indicates that a compound active against [*PSI* ^+^] or [URE3] prions has been spotted, whereas colonies remain white in case of inactive compounds. **C**. Scrapie-infected MovS6 cells were treated for six days with increasing amounts of IQ and then lysed. Cell lysates were then subjected to PK digestion to specifically reveal PrP^Sc^ by immunoblot. The effect of IQ on the steady-state level of PrP (PrP^tot^) was determined on the same MovS6-treated cell lysates in the absence of PK treatment (lower panel). Ratios of Western blot PrP^Sc^/PrP^tot^ signals are indicated below each lane. The blot shown is representative of three independent experiments which all produced similar results.

In order to identify new antiprion compounds, we used the yeast-based screening method we previously described [9,10]. Briefly, [*PSI* ^+^] or [URE3] yeast cells were spread on Petri dishes containing solid agar-based rich medium. Compounds were loaded onto individual filters placed on top of agar surface and dishes were further incubated for three days at 25°C. [*PSI* ^+^] or [URE3] yeast strains form white colonies. Upon the addition of the compound to the filter, compound’s antiprion activity was detected readily and rapidly when a halo of red [*psi* ^-^] or [ure3-0] colonies appeared around the filter. The strength of this method lies on its ability to produce in one simple step, a full gradient of the compounds from active at sub-toxic concentrations to toxic at high concentrations. The BIOMOL’s FDA Approved Drug Library was chosen because it allows drug repositioning. Twelve positive compounds active against [*PSI* ^+^] and [URE3] yeast prions were identified, among which some compounds have previously been described for their antiprion activity, e.g. guanabenz [16] and amiodarone [17]. One of the original hits identified was Imiquimod (1-*iso-*butyl-1H-imidazo[4,5-c] quinolin-4-amine, IQ, Figure **1A**). IQ has potent antiviral and antitumor properties and is used in a large diversity of cutaneous diseases like warts on the skin of the genital and anal areas [18], and is also used to treat actinic keratoses, a skin condition of the face and scalp, and superficial basal cell carcinoma a type of skin cancer [19,20]. IQ does not show a direct activity against the viruses that cause warts [21]. It appears to act via activation of the local innate immune response, through Toll-like receptor 7 (TLR7) stimulation. However its precise molecular mechanism of action remains unclear [22]. IQ displayed a strong activity against [*PSI* ^+^] prion, and was also active against [URE3] yeast prion (Figure **1B**). Then we verified whether IQ was readily curing [*PSI* ^+^] and [URE3] prions and did not interfere with the colorimetric assay [10]. For that purpose, cells from red halos, induced by IQ loading, were streaked onto drug-free YPD medium. They mainly formed red colonies, which emphasized IQ is readily able to eliminate [*PSI* ^+^] and [URE3] yeast prions, similar to the GuHCl positive control (Figure **S1A**).

IQ was then tested for its ability to clear PrP^Sc^ in the mammalian MovS6 cell-based assay [23]. MovS6 cells are murine peripheral neuroglial cell line expressing the ovine PrP gene under the control of its endogenous promoter. These cells are chronically infected by the 127S sheep scrapie prion [23,24] and therefore allows rapid *ex vivo* testing of molecules against mammalian prions. After 6 days of treatment of prion-infected MovS6 cells with a 0 to 20 µM range of concentration of IQ, PrP^Sc^ was detected on the basis of its proteinase K (PK) resistance. For this purpose, a fraction of cell lysates was treated by PK to discriminate PrP^Sc^ proteins, i.e. partially resistant to PK treatment, from total proteins (PrP^tot^). PK treated and untreated cell lysates were separated by SDS-PAGE and immunostained using specific anti-PrP antibodies. Upon IQ treatment, PrP^Sc^ band intensity was greatly reduced, in a dose-dependent manner, with an IC_50_ around 5 µM (Figure **1C**, upper panel). On the contrary, the basal level of PrP^tot^ (PrP^C^ + PrP^Sc^) from cell lysates not treated by PK remained unchanged upon IQ treatment (Figure **1C**, lower panel), indicating that IQ does not act by decreasing the basal level of PrP.

### Activity of IQ in a Mouse Model for Prion Diseases

**Figure 2:**
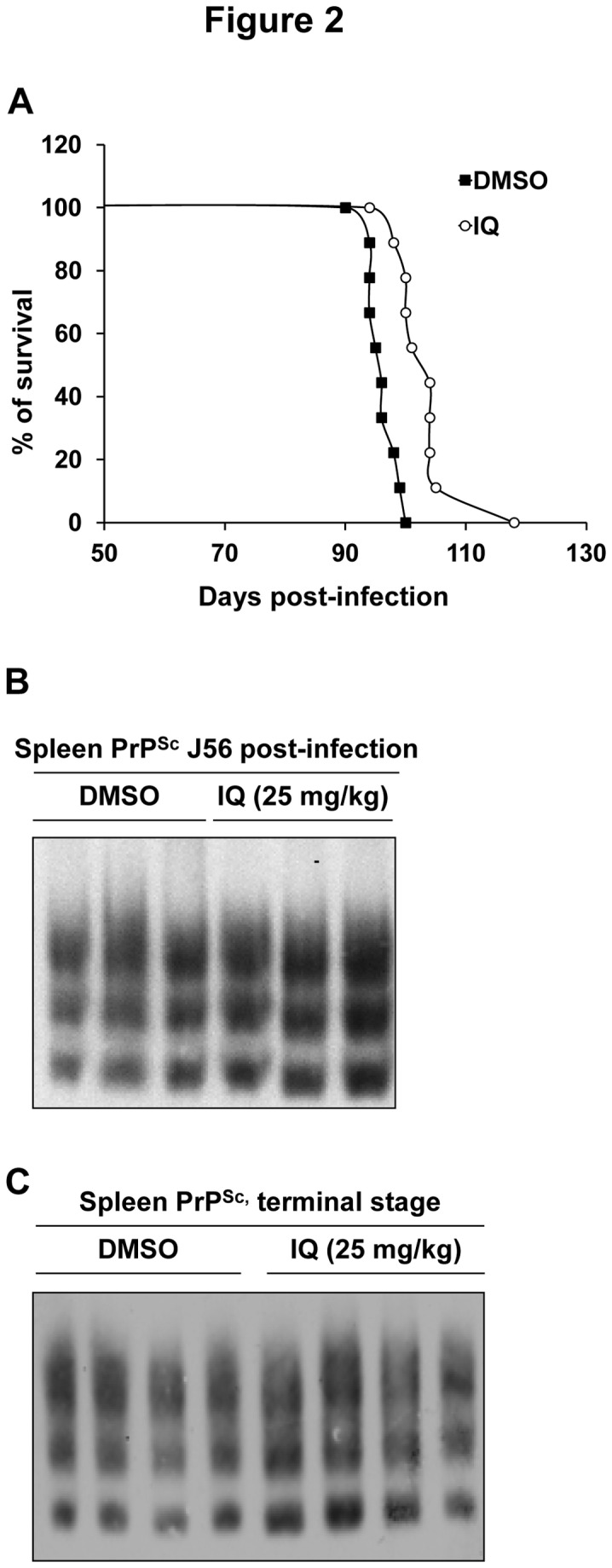
IQ extends survival time in a mouse model for prion diseases. Transgenic mice overexpressing ovine PrP were intraperitoneally infected with scrapie. 12 mice were treated with DMSO (control group) and 12 mice were treated with IQ solubilised in DMSO (IQ group) until the appearance of the first clinical signs in mice from the control group at day 90. **A**. Survival time of mice treated with DMSO (control group) or mice treated with IQ (IQ group). **B**, **C**. Effects of IQ on PrP^Sc^ accumulation in scrapie-infected mouse spleens. The spleen of 3 mice euthanized at 56 days post-inoculation (**B**) and the spleen of 4 mice euthanized at terminal stage (**C**) from both control and IQ groups were analyzed for PrP^Sc^ content. The PrP^Sc^ content of spleens was determined by Western blot. The same amount of spleen tissue proteins was loaded on each well.

We next evaluated the antiprion activity of IQ *in vivo* in a mouse model for prion disease. IQ has been authorized for topical use as the lesions treated are external and bounded, but several *in vivo* trials have previously been performed on various animal species in which IQ exhibited only moderate toxicity (reviewed in 25). To evaluate the *in vivo* potential of IQ against prions, we used transgenic mice expressing ovine PrP [26]. 30 min after the onset of intraperitoneal inoculation of prions, 12 mice were intraperitoneally injected with DMSO (control group) and 12 mice were injected with 25 mg/kg of IQ solubilised in DMSO (IQ group). Mice were then treated 6 days per week for 30 days and then every 3 days from day 31 at the same dose. Mice from both groups were treated until the appearance of the first clinical signs in mice from the control group at day 90, at which time treatment was stopped for mice of both groups. The effect of IQ on mice survival time was assessed on 9 mice per group, as 3 mice from both group were euthanized when still healthy at mid-infection (at 56 days post-inoculation, Figure **2B**) for further analysis of the PrP^Sc^ content of the spleen. IQ treatment was beneficial compared to DMSO treatment, as the mean survival time of the IQ group mice increased by approximately 10%, with a good statistical significance of the difference observed between the two types of regimen (9 mice per group, p<0.0001, Kruskal-Wallis test; Figure **2A**). This effect is quite noticeable given the negative results obtained following treatment of mice with quinacrine (QC) and chlorpromazine (CPZ), two compounds already used for treatment of malaria and psychotic disorders, respectively. Indeed, despite being active against mammalian prion in cell-based assays, QC and CPZ turned out to be inactive both in mouse models for prion diseases and in patients in compassionate treatments of CJD [27,28]. IQ thus presents a significant effect on the survival time of Tg338 mice, an animal model that might be difficult to cure, due to the high infectious load inoculated and to the high level of expression of PrP. We expect that IQ effects should be more noticeable in a less stringent model [29]. PrP^Sc^ accumulation in the spleen was assessed in mice euthanized at mid-infection (3 mice per group) (Figure **2B**) and at terminal stage (4 mice per group) (Figure **2C**) [26]. IQ did not affect significantly spleen PrP^Sc^ level, although it showed a beneficial effect on mouse survival, suggesting that IQ may delay 127S prion neuroinvasion phase. Hence, this study suggests a potential novel therapeutic indication of imidazoquinolines compounds such as IQ for the treatment of prion-based diseases in mammals, including humans.

### A new synthesis pathway for IQ and its chemical derivatives

**Figure 3:**
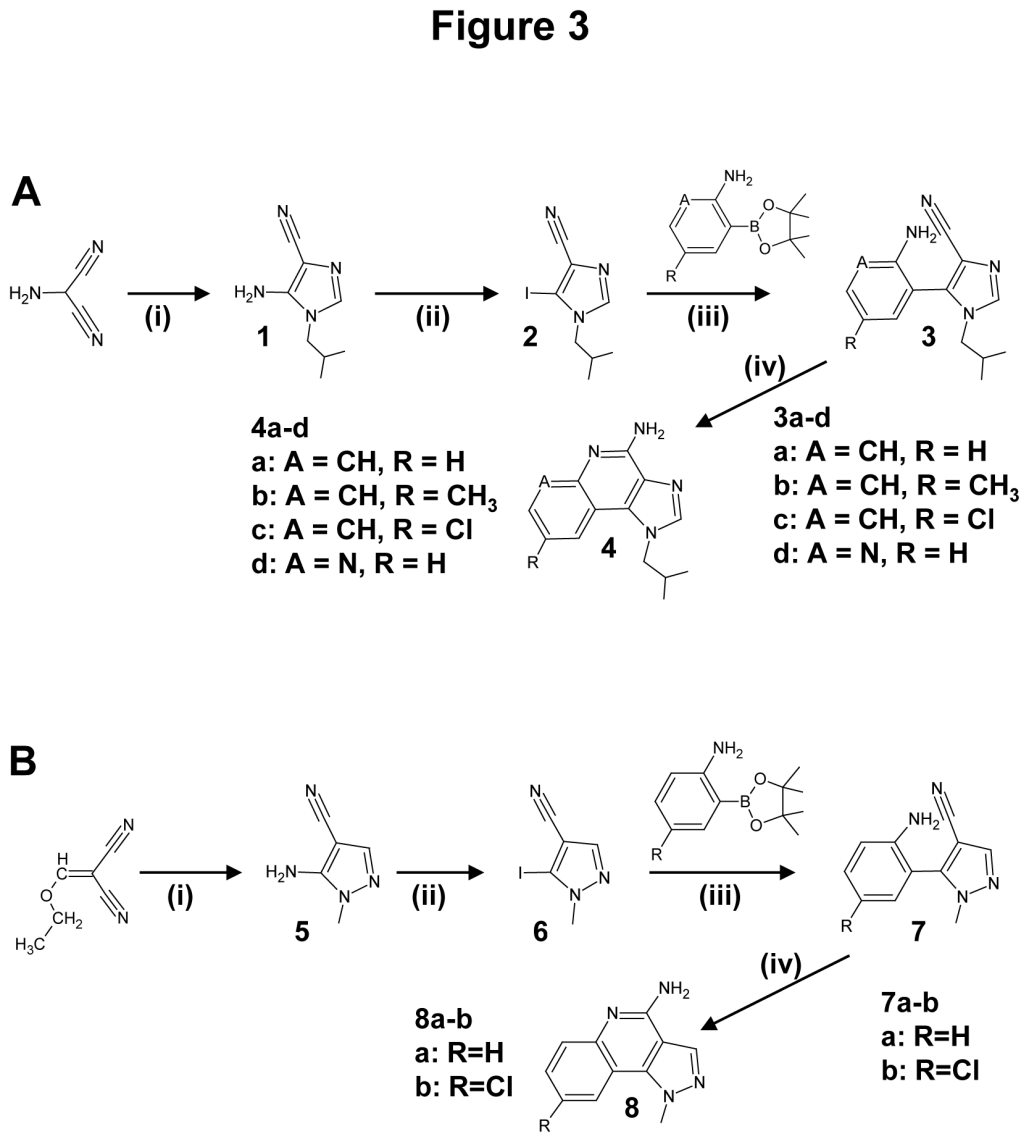
Reagents and conditions for the preparation of IQ and its chemical derivatives. **A**. (**i**) Step 1: CH(OEt)_3_, CH_3_CN 30 min reflux; Step 2: (*iso-*butylamine) 12h 20°C; (ii) CH_2_I_2,_ *iso-*pentylnitrite; (iii) 2N Na _2_CO_3_, Pd[(P(C_6_H_5_)_3_]_4_, dioxane**; (iv)** NaNH_2_, toluene, 2h at 80 °C. **B**. (**i**) CH_3_NHNH_2_, NEt_3_, THF, 60 °C; (**ii**) CH_2_I_2_, *iso*-pentylnitrite; (**iii**) 2N Na _2_CO_3_, Pd](P(C_6_H_5_)_3_]_4_, dioxane, 80 °C; (iv) NaNH_2_, toluene.

IQ belongs to the imidazoquinolines class of bioactive compounds. It is commercialized under different brand names (Aldara®, Zyclara® and Beselna®). The imiquimod series continues to be the subject of intensive drug discovery studies in regard to the broad therapeutic potencies of immune enhancers. IQ contains an imidazoquinoline moiety which is found in other biologically active compounds such as modulators of the A3 adenosine receptor [30] and anticancer drugs used for the treatment of melanomas [31]. IQ analogues such as Gardiquimod, R-848 and Loxoribine have recently entered clinical trials. Several routes to synthesize IQ have been previously reported and patented. The simplest one was proposed by V. Nakkada [32]. Briefly, the process consisted of heating of 4-chloro-1-isobutyl-1H-imidazole[4,5-c] quinoline with formamide. In most of the previously described methods, the imidazole ring is formed during the last steps of the process precursered by quinolines derivatives. In the presented method, the central pyridine ring of the tricyclic system is formed during the last step. This new simple synthesis route allows to obtain IQ and other aminoimidazoquinolines, as well as structurally related amino pyrazoloquinoline by allowing a Suzuki-Miyaura coupling between a 2-aminoarylboronic ester and a iodocyanoimidazole (Figure **3**). The biarylic intermediate was easily converted into a tricyclic aromatic compound under basic conditions. This method, a model for short synthesis pathway of IQ (Figure **3A**), was further extended to pyrazoloquinolines (Figure **3B**). The cyanoaminoimidazole **1** was obtained using a known procedure [33]: Aminomalononitrile was reacted with triethylformate followed by the addition of iso-butylamine (Figure **3A**). In the second step, the iodination was achieved using diiodomethane containing *Iso-*amylnitrite (*iso*-pentylnitrite) leading to iodoimidazonitrile **2** (Figure **3A**). The Suzuki coupling was then performed under classical reaction conditions using ortho-aminoboronic acid to obtain birarylaminonitriles **3**. Several approaches were investigated for the final cyclisation step. It was efficiently achieved upon heating sodium amide in toluene. The final compounds **4** could be isolated as free bases or hydrochlorides salts (Figure **3A**). The same approach was also used for the synthesis of the previously unknown amino pyrazoloquinolines. Aminopyrazolonitrile **5** can be efficiently prepared by the reaction of hydrazines with ethoxymethylene aminonitrile (Figure **3B**). They were converted into the corresponding iodopyrazolonitrile **6** under the same conditions as for the synthesis of **3**. The coupling of **6** with 2-aminoboronic ester led to the intermediate biaryl **7** which was then converted into aminopyrazolo[4,3-c] quinolines. The new synthesis pathway described here now allows easy synthesis of IQ and efficient synthesis of IQ derivatives to date hard to achieve.

### Activity of chemical derivatives of IQ

**Figure 4:**
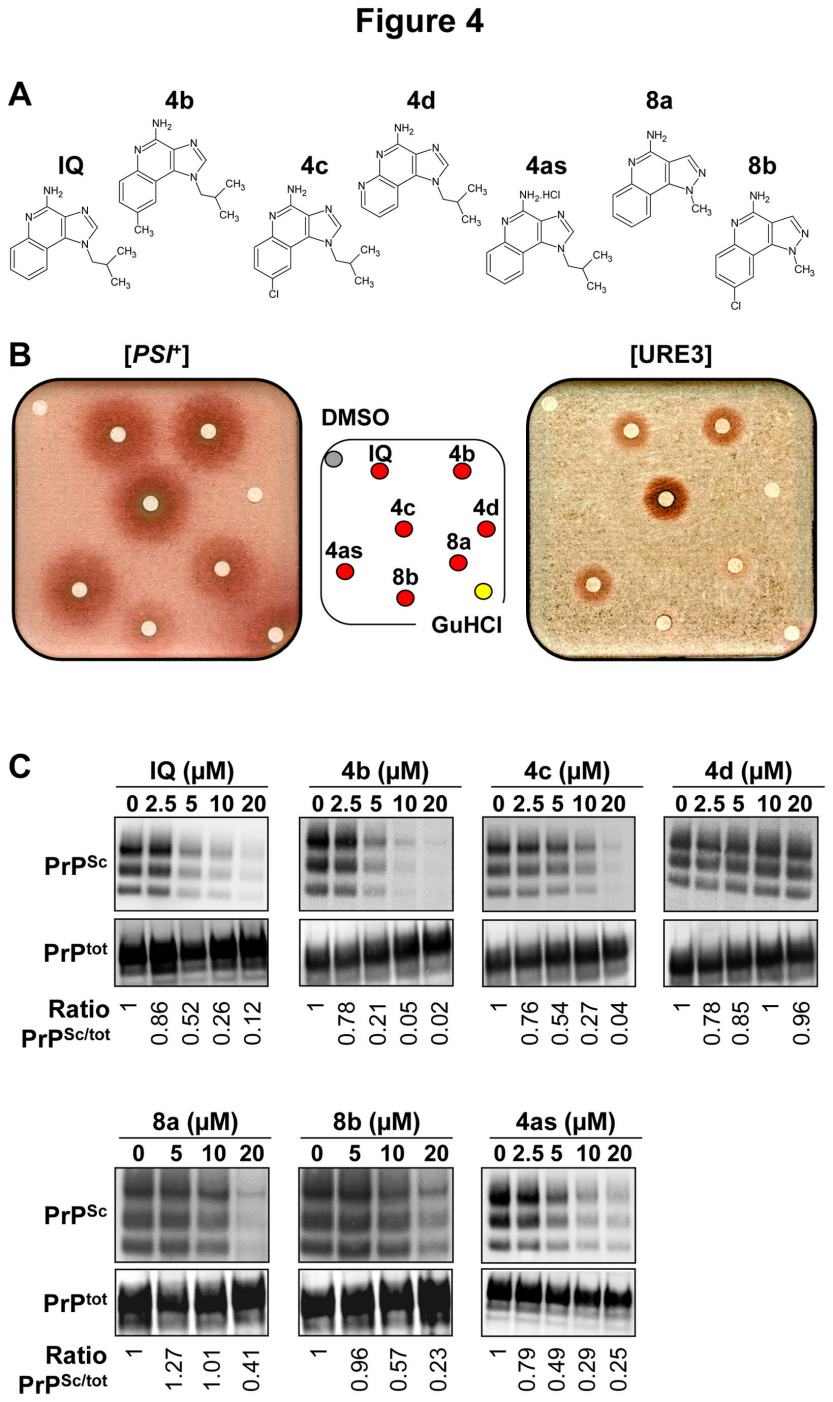
Activity of the chemical derivatives of IQ. **A**. Chemical structures of the 5 derivatives of IQ. **4as** corresponds to the hydrochloride of IQ. **B**. STRg6 [*PSI* ^+^] and SB34 [URE3] strains were spread on YPD medium supplemented with 200 and 800 µM GuHCl, respectively. 50 nmoles of IQ and of its 5 chemical derivatives were spotted on filters as described in Figure **1B**. The top left filter correspond to DMSO and the bottom right filter was spotted with GuHCl (positive control). **C**. Activity of IQ and its chemical derivatives against PrP^Sc^ in the MovS6 cell-based system described in Figure **1C**. Ratios of Western blot PrP^Sc^/PrP^C^ signals are indicated below each lane. The blot shown is representative of three independent experiments which all produced similar results.

The new synthesis pathway described above enabled the production of a first set of IQ chemical derivatives, i.e. a first corpus of structure-activity relationship (SAR). The five original chemical derivatives of IQ obtained (Figure **4A**) were tested against both yeast and mammalian prions using respectively the yeast-based ([*PSI* ^+^] and [URE3] prions) and MovS6-based assays described above (Figure **4B & 4C**). We also checked that the red halos induced by IQ’s chemical derivatives corresponded to [*PSI* ^+^] and [URE3] cured cells (Figure **S1B**). This structure/activity relationship study allowed the identification of four active (**4b**, **4c**, **8a, 8b**) as well as one inactive (**4d**) derivatives which constitutes a valuable negative control (Figure **4B & 4C**). Interestingly, activity of the various derivatives of IQ in the MovS6 cell-based assay paralleled their activity against yeast prions. For example, the replacement of a phenyl or substitution of phenyl by a pyridine completely abolished the antiprion activity (**4d**) whereas a methyl in position 8 was associated with a slight increase of antiprion activity (4b) (Figure **4B & 4C**). On the contrary, a chlorine group in the same position slightly reduced the antiprion activity of the bioactive compounds (**4c** & **8b**). These parallels in SAR in both yeast and mammalian cells strongly support the idea that these drugs target a conserved pathway.

### Antiprion activity of IQ does not involve its agonist activity on Toll-like receptors 7/8

**Figure 5:**
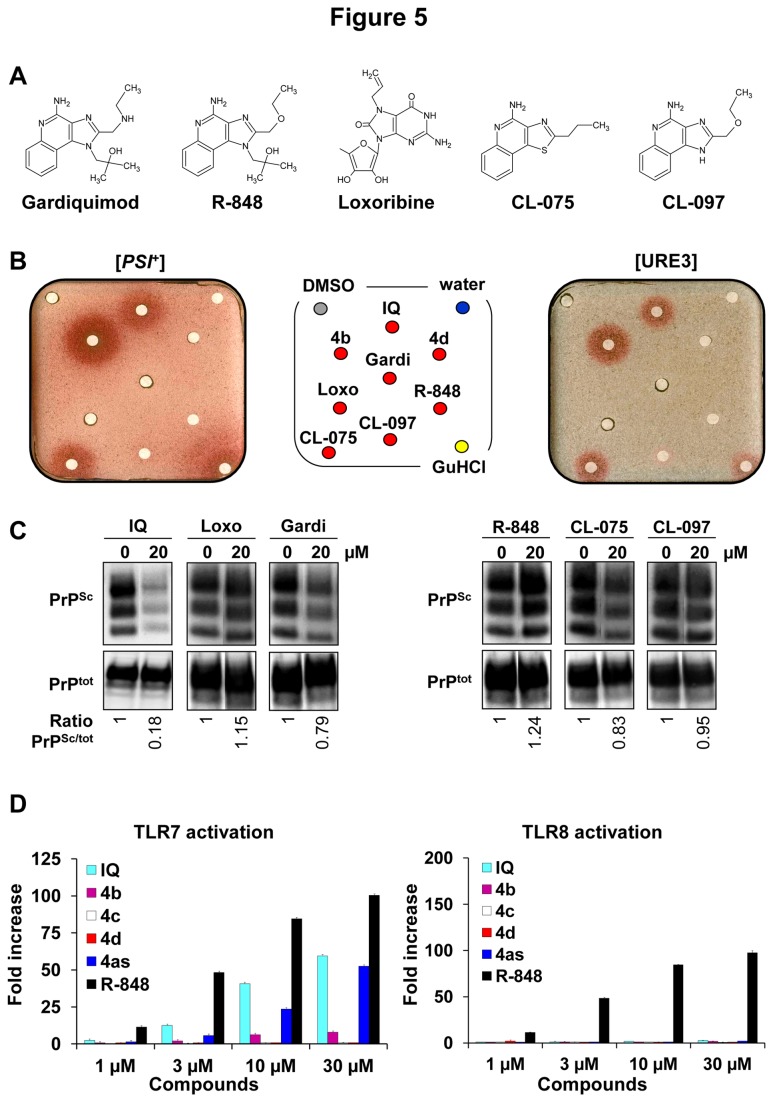
Antiprion activity of some TLR7/8 agonists related to IQ. **A**. Structures of Gardiquimod, R-848, Loxoribine, CL-075 and CL-097, five other known TLR7/8 agonists. **B**. These five TLR7/8 agonists were tested against both [*PSI* ^+^] and [URE3] yeast prions (50 nmoles of compounds per filter, 5 nmoles for CL-075) as described in Figure **1B**. **C**. Evaluation of the activity of Gardiquimod (Gardi), R-848, Loxoribine (Loxo), CL-075 and CL-097 against mammalian prion using the MovS6 cell-based assay. Ratios of Western blot PrP^Sc^/PrP^tot^ signals are indicated below each lane. The blot shown is representative of two independent experiments which all produced similar results. **D**. The potential of IQ chemical derivatives 4b, 4c and 4d as TLR7 (left panel) and TLR8 (right panel) agonists was also evaluated *ex vivo*. Briefly, 293XL‐hTLR7 and 293XL‐hTLR8 cells transiently transfected with an NF-κB inducible reporter plasmid were incubated with compounds at indicated concentrations and Luciferase production, which reflects the activation of the receptor, was quantified. Experiments were performed two (TLR8) or three (TLR7) times in duplicates.

In order to determine if the antiprion activity of IQ and its chemical derivatives could be linked to their agonist activity on Toll-like receptors (TLRs), we first tested the potential antiprion activity of commercially available TLR7 and TLR8 agonists which are pharmacologically and chemically close to IQ such as Gardiquimod, Loxoribine and R-848 (TLR7 agonists), and CL-075 and CL-097 (TLR7/8 agonists) (Figure **5A**). Indeed, like IQ, these TLR7 or TLR7/8 agonists are used in various preclinical tests to stimulate the innate and acquired immune responses. As shown in Figure **5B**, only one of the five TLR7/8 agonists tested, CL-075, exhibited some antiprion activity against both [*PSI* ^+^] and [URE3] yeast prions. CL-097 was slightly active only against [URE3]. In addition, only Gardiquimod and CL-075 displayed a very modest activity against PrP^Sc^ in MovS6 cells (Figure **5C**). Next, the capacity of IQ and its chemical derivatives **4b** and **4c** to activate TLR7 and TLR8 was evaluated. As shown on the left panel of Figure **5D**, IQ/**4as** and the TLR7/8 agonist R-848 activated cells through TLR7 whereas **4b**, **4c**, and **4d** had no agonist activities on TLR7. Contrary to R-848, neither IQ nor **4as**, **4b**, **4c**, and **4d** were able to activate TLR8 receptor (Figure **5D** **, right panel**). Thus the antiprion activity of IQ and its chemical derivatives does not parallel their agonist activity on TLR7/8. In addition, the antiprion activity of IQ against *S. cerevisiae* prions cannot be due to its agonist activity on TLR7 since yeast does not contain this type of receptors. Altogether, these results suggest that the antiprion activity of IQ does not involve the same mechanism than the one responsible for its capacity to trigger immune responses.

Recent data suggested that IQ could exert its effect by increasing the level of the opioid growth factor receptor (OGFr) [34]. Our data showed that the antiprion activity of IQ and its chemical derivatives is independent from their ability to stimulate TLRs. This provides another example where IQ’s beneficial effects may be independent of its capacity to trigger the innate immune system.

### IQ and its chemical derivative 4b inhibit the protein folding activity of the ribosome

**Figure 6:**
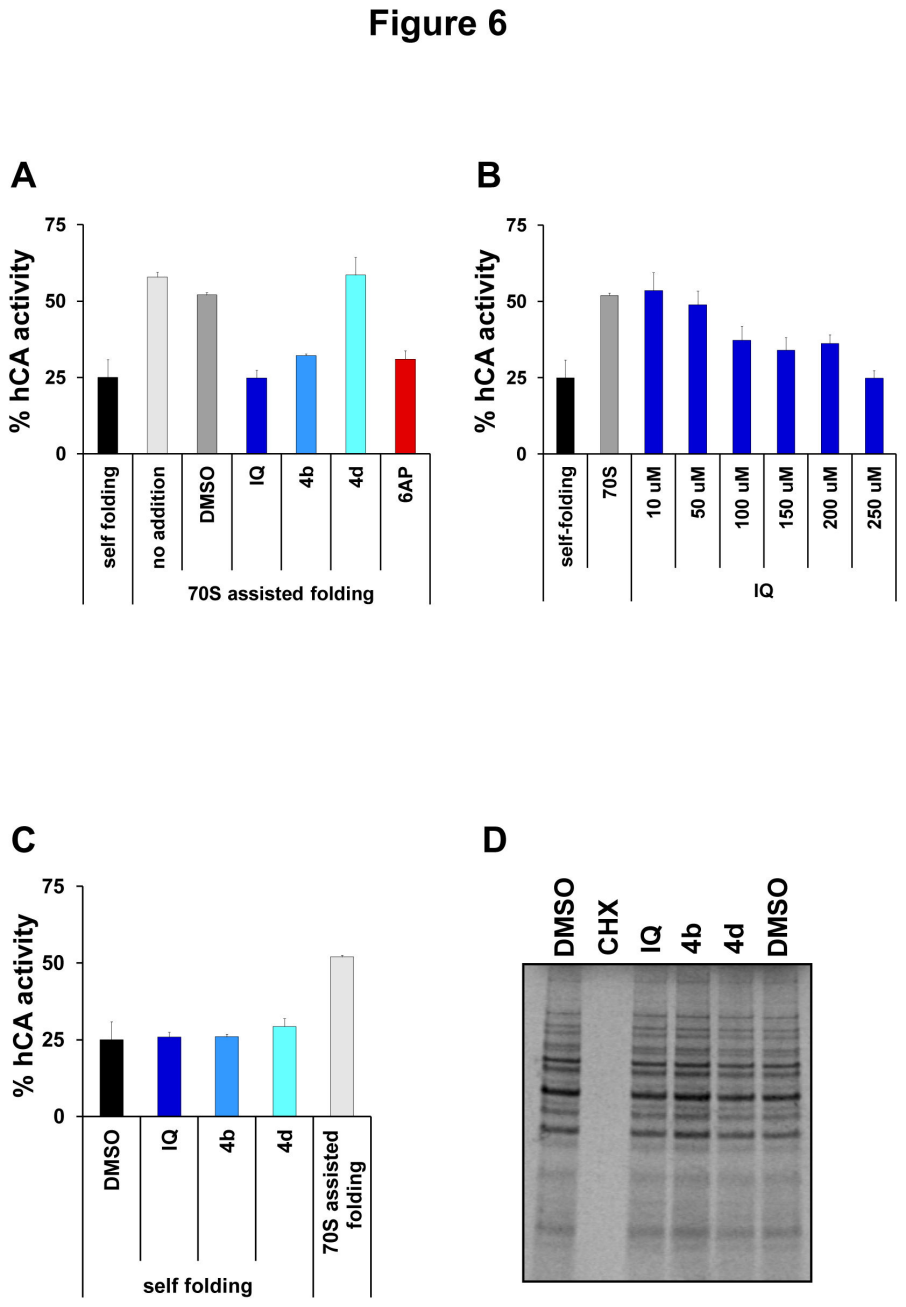
*in vitro* specific anti-PFAR activity of IQ and its chemical derivative 4b. **A**. The effect of the various drugs on ribosome-assisted folding of GuHCl-denatured hCA was evaluated. Correct refolding was assessed by measuring the recovery of hCA enzymatic activity as a function of time in comparison to that of the native enzyme stored undiluted on ice (normalized to 100%). Self-refolding of hCA was about 25%, the refolding with 70S ribosome increased to 55% due to PFAR. In the presence of 250 µM of IQ and 4b, PFAR was fully inhibited, whereas 250 µM 4d showed no inhibitory effect. 250 µM 6AP was used as a positive control [11]. **B**. IQ inhibits PFAR in a dose-dependent manner. **C**. IQ and its chemical derivatives (250 µM) do not affect the self-folding of GuHCl-denatured hCA. **D**. The effect of the indicated compounds (100 µM) on global *in vivo* translation in living yeast cells was evaluated. Briefly, various drugs or DMSO alone were added to yeast cells in exponential growth in YPD rich medium at a final concentration of 100 µM. After 20 minutes, radiolabelled [^35^S] methionine/cystein was added for 20 minutes. The cells were lysed and analyzed by SDS-PAGE followed by autoradiography. Error bars correspond to standard deviations.

IQ is structurally quite close to 6AP, an antiprion compound we engineered in the course of a structure/activity relationship study we led around phenanthridine [9]. 6AP is a specific inhibitor of the protein folding activity of the ribosome (PFAR), which is an RNA-based activity borne by the domain V of the large rRNA of the large subunit of the ribosome [13,35–39]. This activity of the ribosome is still poorly understood but is preserved throughout evolution, from prokaryotes to eukaryotes. Since IQ antiprion activity is not linked to its TLR7 agonist activity, we wondered if this drug might, like 6AP, modulate PFAR. Human Carbonic Anhydrase (hCA) was used as a substrate for *in vitro* assisted folding experiments. Denatured hCA was diluted in native buffer either alone (to determine self-folding efficiency) or in the presence of preparations of *E. coli* 70S ribosome. The correct refolding of hCA was assessed by following the reappearance of its enzymatic activity in comparison to the native hCA. Self-folding restored about 25% of hCA activity. PFAR borne by *E. coli* ribosomes restored about 55% of hCA activity in the absence or presence of DMSO (Figure **6A**). In the presence of IQ and its chemical derivative **4b**, the protein folding activity of 70S ribosomes was greatly inhibited (Figure **6A & 6B**) whereas the self-folding of hCA was not affected (Figure **6C**). This inhibition was specific since **4d** compound, which presented no antiprion activity, also showed no inhibition of PFAR (Figure **6A**). As the main function of the ribosome is protein synthesis, the effect of IQ and **4b** on translation was also assessed *in vivo* in yeast. None of the tested compounds exhibited a significant effect on global protein synthesis, in contrast with cycloheximide (CHX), a known inhibitor of global translation, which completely inhibited protein synthesis at the tested concentration (Figure **6D**). Therefore, at concentrations at which they exhibit antiprion activity, neither IQ nor **4b** affected protein synthesis *in vivo* in yeast whereas, like 6AP [11], they are specific inhibitors of the protein folding activity of the ribosome. Taken together, these results indicate that IQ and **4b** are specific inhibitors of PFAR, and that their ability to inhibit PFAR paralleled their antiprion activity. They also confirm that PFAR might be involved in prion propagation both in yeast and mammals. Our recent data confirm that PFAR is involved in prion propagation in yeast (CV, PN, FS & MB unpublished data). In addition to their potential therapeutic application, 6AP, GA and IQ are currently the sole PFAR specific inhibitors. They can be regarded as precious tools for explaining the biological role of PFAR which has only been partially explored to date, in particular, in prion diseases, and more generally in amyloid-based diseases [13].

## Materials and Methods

### Yeast strains and culture media

Yeast strains used in this study were STRg6 (74-D694, Mata, *erg6::TRP1*, *ade1-14*, *trp1-289*, *his3Δ200*, *ura3-52*, *leu2-3,112*, [*PSI* ^+^]) and SB34 (Mata, *erg6::TRP1*, *dal5::ADE2*, *ade2-1, trp1-1*, *leu2-3,112*, *his3-11,15*, *ura2::HIS*, [URE3]) and were grown and used as previously described [9].

### Commercially available compounds

BIOMOL’s FDA-Approved Drug Library (Enzo Life Sciences) is a collection of 640 FDA-approved molecules all in use in the clinics selected to maximize chemical and pharmacological diversity. The compounds are supplied as 2 mg/ml DMSO solutions. IQ and GuHCl were purchased from Sigma Aldrich. CL-075, CL-097, Gardiquimod and Loxoribine powders were purchased from InvivoGen. R-848 was purchased from IMGENEX (1 mg/ml in water).

### Yeast-based antiprion screening assay

This assay was performed as previously described [9,10]. Briefly, an aliquot of an exponentially growing culture (340 µl of 0.55 OD_600_ culture of STRg6 or 170 µl of 0.55 OD_600_ culture of SB34 strains) was spread homogeneously using sterile glass beads on square plates (12 x 12 cm) containing YPD solid medium supplemented with 200 µM (for [*PSI* ^+^] STRg6 strains) or 800 µM Guanidine hydrochloride (for SB34 [URE3] strain). Small sterile filters (Thermo-Fisher) were then placed on the agar surface and individual compounds were applied to each filter. DMSO, the vehicle compound, was applied to the top left filter as a negative control, and 10 µl of 300 mM GuHCl solubilized in DMSO was applied to the bottom right filter as a positive control. 2 µl of each of the 640 compounds of the BIOMOL chemical library, or 50 nmoles of IQ chemical derivatives 4b-d, or 50 nmoles of TLR7 and TLR7/8 agonists Gardiquimod, R-848, Loxoribine and CL-097, or 5 nmoles of CL-075 were applied on each of the remaining filters. Plates were then incubated 5 days at 25°C and scanned using a Snap Scan121s2 (Agfa).

### PrP^Sc^ clearance assay in MovS6 cells

Experiments were performed as previously described [23]. Briefly, MovS6 cells chronically infected with ovine 127S prion strain were treated for six days with the indicated concentrations of compounds and then lysed (0.5% Na deoxycholate, 0.5% Triton X-100, 5 mM Tris-HCl pH 7.4). To distinguish between cellular (PrP^C^) and pathological forms of PrP^Sc^, 250 µg of cell lysates were digested by proteinase K (PK) as PrP^Sc^ is partially resistant to proteolysis. The detection of PrP^tot^ was performed on 25 µg of crude cell lysate. Proteins were analyzed by 10% SDS-PAGE (Invitrogen) and transferred to 0.45 µm nitrocellulose membranes (Whatman). Membranes were incubated for two hours with 1:40 000 anti-PrP antibody (Sha31, Bertin pharma). The membranes were then washed with fresh PBS 1X / 0.1% Igepal and incubated for 45 min with 1:3000 secondary HRP-conjugated antibodies (Biorad), and analyzed by Enhanced Chemiluminescence (GE Healthcare) using a Vilber-Lourmat Fusion SL image acquisition system which allows precise quantification of the signals.

### Ethics Statement

Animal experiments were carried out in strict accordance with EU directive 2010/63 and were approved by the author’s institution local ethics committee (Comethea, INRA Agroparitech ethics committee, permit number 12/034). All efforts were made to minimize suffering.

### Mouse model for prion-based disease

Experiments were performed as previously described [40]. 24 mice overexpressing ovine PrP (tg338 line [24]) were infected intraperitoneally with 100 µl of the 127S scrapie strain at 0.02% (w/v) dose. This strain has an intracerebral infectious titre of 10^9^ ID_50_ U/g of brain [24]. 12 mice (IQ group) were then intraperitoneally injected with a dose of 100 µl of 2.5 mg/ml IQ solubilized in 8% DMSO (25 mg/kg) 30 min after 127S injection, and 12 infected mice were intraperitoneally injected with 100 µl of 8% DMSO (control group). Mice were then treated 6 days per week for 30 days and then every 3 days from day 31 at the same dose. The treatment was stopped around day 90 post-infection, when the first symptoms appeared in mice from the control group. Mice were euthanized at terminal stage according to ethics rules. At mid-treatment (56 days post-infection), 3 of 12 mice from both control and IQ groups were euthanized. Spleens were also collected from 4 mice euthanized at terminal stage of disease. Spleens collected at mid-treatment and at terminal stage of disease from euthanized mice were analyzed for PrP^Sc^ content, as previously described [2,16]. Briefly, spleen tissues were homogenized at 20% (w/v) in 5% glucose with a Rybolyser (Hybaid). PrP^res^ was extracted according to the Biorad test protocol, by using 200 mg/ml PK for 10 min. at 37°C. After denaturation in Laemmli buffer, proteins were analyzed by SDS-PAGE (Invitrogen) and transferred to nitrocellulose membranes and immunoblotted with 0.1 mg/ml Sha31 anti-PrP antibody. The equivalent of 1 mg of spleen tissue was loaded onto the gels. Immunoreactivity was visualized by chemiluminescence (GE Healthcare).

### Chemical Methods

The synthesis methods for the compounds **1**, **2**, **3a**, **3b**, **3c**, **3d**, **4a**, **4b**, **4c**, **4d**, **5**, **6**, **7a**, **7b**, **8a** and **8b** are described in Methods S1.

^1^H-NMR, ^13^C-NMR spectra, IR spectra and microanalyses are available upon request.

### TLR7 and TLR8-based assays

293XL‐hTLR7 cells and 293XL‐hTLR8 cells (Invivogen) were transiently transfected with an NF-κB inducible reporter plasmid (Clontech). On day 0, cells were seeded overnight at 5.10^5^ cells/mL on 6 well plates. On day 1, cells were transfected for 6 hours by pNFkB‐luc plasmid using FuGENE® 6 Reagent (Roche) as recommended by the manufacturer. Transfected cells were further incubated overnight at 37°C, followed by a 24h incubation with different compounds at the indicated concentrations. The presence of Luciferase was revealed using Steady Glo Luciferase assay system (Promega). The light emitted by Luciferase activation in each well was quantified as “Count Per Second” (CPS) on a Victor apparatus (Perkin-Elmer). Fold increase was quantified as the ratio between experimental CPS (stimulus) and spontaneous CPS (medium).

### Translation assay

A yeast culture grown at 29°C in YPD (OD_600 nm_ = 0.6 in exponential phase of growth) was incubated with the indicated compounds (100 µM) or the corresponding volume of DMSO for 20 min at 29°C at which time [^35^S] methionine and cystein were added for 20 min (PerkinElmer Life Sciences). Cells were then harvested and lysed (lysis buffer: 25 mM Tris-HCl pH 7.4, 100 mM NaCl, 0.2% Triton X-100, antiproteases cocktail (Roche), 1 mM phenyl-methylsulfonyl fluoride). Crude extracts were analyzed by 10% SDS-PAGE (Invitrogen). The gel was dried and analyzed using a Typhoon 9400 Phosphorimager (GE Healthcare).

### In vitro ribosome assisted protein folding assay

70S *E. coli* ribosomes were prepared using sucrose gradient zonal ultracentrifugation as described previously [41]. For the *in vitro* refolding experiments described in [15], human Carbonic Anhydrase (hCA) at a concentration of 30 µM was denatured by an overnight treatment at room temperature with 6 M GuHCl and 30 nM EDTA. To allow refolding, hCA was diluted 100 times at a final concentration of 300 nM in a buffer containing 20 mM Tris-HCl (pH 7.5), 100 mM NaCl and 5 mM magnesium acetate for 30 min with or without 70S ribosomes at a final concentration of 300 nM, in the absence or presence of compounds. The refolding of hCA, as a function of the native enzyme activity (normalized to 100%), was followed by a colorimetric assay measuring the increase of OD_400_ with time when hCA substrate, para-nitrophenyl acetate (pNPA), was added directly to the refolding mix at a final concentration of 500 µM. To check if drugs may affect hCA self-folding, denatured hCA was diluted 100 times at a final concentration of 300 nM in a buffer containing 20 mM Tris-HCl (pH 7.5), 100 mM NaCl and 5 mM magnesium acetate for 30 min in the presence of compounds, in the absence of 70 ribosomes.

## Supporting Information

Methods S1 (DOC)

Figure S1 **Cells from red halos surrounding filters on which IQ (A) or IQ, 4b, 4c, 4d, 8a, 8b (B) was loaded were streaked on drug-free YPD medium.** Cells surrounding filters on which DMSO and GuHCl were loaded were used as negative and positive controls, respectively.
(TIF)
