# Supplementary material for: The Toll-Like Receptor Agonist Imiquimod Is Active against Prions
Source: PLoS One. 2013 Aug 16;8(8):e72112. doi: 10.1371/journal.pone.0072112 (PMC3745460; doi:10.1371/journal.pone.0072112)
Supplement: Methods S1 — (DOC) [file pone.0072112.s001.doc]

**SUPPLEMENTARY MATERIAL**

**Chemical Methods.**

Reactions were monitored by TLC using Merk silica gel 60F-254 thin layer plates. Column chromatography experiments were carried out on SDS Chromagel 60 ACC, 40-63 m. Column chromatography (CC) was performed using silica gel (70-200 μm). Melting points were determined on a kofler hot-stage (Reichert) and are uncorrected. NMR spectra were recorded on Bruker Avance 400 MHz. Chemical shifts are given in ppm downfield of tetramethylsilane (TMS) used as an internal standard. Infrared spectra were recorded on a FTIR Schimadzu 8300. The HPLC analyses were carried out on a system consisting of a Waters 600 system controller, a Jones Chromatography heater-chiller oven and a Waters 994 photodiode array detector. Mass spectra (electrospray ionization (ESI) was measured on a TSQ Quantum (Thermo Electron Corporation) instrument. Elemental analyses for C, H and N were performed on a Thermoquest EA-110 apparatus.

**5-Amino-1-(2-methylpropyl)imidazole-4-carbonitrile** (**1**). Anhydrous ammonia was passed, at room temperature, through a stirred suspension of 2-aminomalononitrile tosylate (10 g, 39 mmol) in 200 mL CH_3_CN. After 45 min, ammonia was stopped and the solution was cooled at 15 °C. The solid fraction was filtered off and washed once with 10 mL CH_3_CN. The filtrate was combined with the washing and concentrated to half of its volume. To this solution, triethyl orthoformate (6.6 mL, 39.5 mmol) was added and the solution was refluxed 30 min. After cooling to 20 °C, 2-methylpropylamine (*iso*-butylamine) (3.95 mL, 39.5 mmol) was added and the mixture was stirred overnight at room temperature. The solution was evaporated to dryness *in vacuo* and the residue was partitioned between CH_2_Cl_2_ and 2N Na_2_CO_3_. The organic layer was evaporated and the residue was triturated in 20 mL AcOEt. The brown crystals that formed were separated and could be used for the next step.

Yield: 50-60 %. An analytical sample was prepared by crystallisation from AcOEt. Mp: 193-195°C. ^1^H-NMR (CDCl_3_) δ 0.91 (d, *J =* 6.5 Hz, 6H, 2CH_3_); 1.98 (non, 1H, CH); 3.50 (d, 2H, CH_2_); 3.83 (brs, 2H, NH_2_); 6.97(s, 1H, imidazol). ^13^C-NMR 20.24; 29.38; 51.96; 116.11; 113.89.

**5-Iodo-1-(2-methylpropyl)imidazole-4-carbonitrile** (**2**). Isoamylnitrite (isopentylnitrite) (4.68 g, 40 mmol) was added dropwise over 30 mn to a hot (80°C) suspension of compound **1** (3.28 g, 20 mmol) in CH_2_I_2_ (100 mL). Stirring was pursued 1 h at the same temperature. Diiodomethane was then recovered as a pink oil by distillation *in vacuo* Bp(10 mm): 90-100 °C***.*** The residue was taken up with AcOEt and crystallized upon trituration with Et_2_O.

Yield: 80-85 %. Mp: 174-176°C. ^1^H-NMR(CDCl_3_) δ 0.75(d, 6H, 2 CH_3_); 1.9­6 (non, 1H, CH); 3.55 (d, 2H, CH_2_); 7.42 (1H, s, imidazol). ^13^C-NMR δ 19.31; 29.15; 55.76; 82.30; 114.23; 122.26; 140.93. IR(KBr) ν 763; 1010; 1226; 1373; 1496; 2229; 2360; 2962; 3118; 3564; 3618 cm^-1^.

**5-(2-Aminophenyl)-1-(2-methylpropyl)imidazole-4-carbonitrile** (**3a**). A slow bubbling of nitrogen was maintained throughout the reaction. Pinacol 2-(aminophenyl)boronate (2.80 g, 10 mmol) was added to a solution of Pd[(P(C_6_H_5_)_3_]_4_ (0.55 g, 0.5 mmol) in 10 mL 2N Na_2_CO_3_ and 20 mL dioxane. The mixture was stirred for 2h at 80 °C. After cooling to 20 °C the mixture was concentrated *in vacuo* to half of its initial volume and then extracted twice with 20 mL AcOEt. The organic layer was dried over Na_2_SO_4_ and evaporated. The residue was triturated with 2 mL AcOEt to afford **3**. Yield: 85-95 %. Mp: 145-150°C. ^1^H-NMR(CDCl_3_) δ 0.78 and 0.80 (2d, 2x3H, *J* = 6.5 Hz, 2 CH_3_) ; 1.80(non, 1H, CH) 3.60(brs, 2H, NH_2_); 3.65(d, 2H, CH_2_); 6.80(d, 1H, *J*= 7 Hz, Aro); 6.83(t, 1H, Aro); 7.30(t, 1H, Aro) ; 7.60(s, 1H, imidazol). IR(KBr)ν 748; 1449; 1643; 2229; 2955; 3440; 3741 cm^1^.

**5-(2-Amino-5-methyl-phenyl)-1-isobutyl-imidazole-4-carbonitrile** (**3b**) 0.78(d, *J* = 6.5 Hz, 6H, 2 CH_3_); 1.83(non, 1H, CH(CH_3_)_2_); 3.68(m, 2H, CH_2_); 3.75(brs, 2H, NH_2_).

**5-(2-Amino-5-chloro-phenyl)-1-isobutyl-imidazole-4-carbonitrile** (**3c**) ^1^H-NMR(CDCl_3_) δ 0.78(d, *J* = 6.5 Hz, 6H, 2 CH_3_); 1.83(hept, 1H, CH(CH_3_)_2_); 3.68(m, 2H, CH_2_); 3.75(brs, 2H, NH_2_); 6.75(d, 1H, *J* = 7.0 Hz, 7.07(s, 1H, Aro); 7.28(d, 1H, Aro); 7.61(s, 1H, imidazol). IR (KBr) ν 640; 794; 1253; 1581; 1643; 2341; 2761; 2869; 2962; 3105; 3178; 3305; 3673; 3749; 4463 cm^-1^.

**5-(2-Amino-3-pyridyl)-1-isobutyl-imidazole-4-carbonitrile** (**3d**) ^1^H-NMR(400 MHz, CDCl_3_) δ 0.81(d, *J* = 6.5 Hz, 6H, 2 CH_3_); 1.86(hept, 1H, CH(CH_3_)_2_); 3.72(m, 2H, CH_2_); 3.90 (brs, 2H, NH_2_); 7.17(t, J = 8 Hz, 1H, pyridyl); 7.38(d, 1H, pyridyl ); 7.69(s, 1H, imidazol), 8.12(d, 1H, pyridyl)

**4-Amino-1-(2-methylpropyl)-imidazo-[4,5-c]quinoline (Imiquimod) (4a).** NaNH_2_ (0.2 g, 5 mmol) was added under N_2_ at room temperature to compound **3** (1.20, 5 mmol) suspended in 20 mL of anhydrous toluene. The mixture was stirred at 90-100 °C. Monitoring of the reaction by thin layer chromatography showed complete conversion in less than 2h. After cooling below 5°C, 2 mL of ice water were added dropwise and the mixture was evaporated until dryness (**4a**). Imiquimod was isolated upon trituration of the residue with 5 mL of cold water. Yield: 90-96%. Mp > 260°C. ^1^H-NMR (400 MHz, CDCl_3_) 1.02(d, *J* = 6.5 Hz, 6H, 2CH_3_); 2.35(non, 1H, CH); 4.30(d, 2H, CH_2_); 5.50(brs, 2H, NH_2_); 7.32(t, *J*= 8.5 Hz, 1H, 8-H), 7.55(t, 1H, 7-H); 7.80(s, 1H, 2-H); 7.82(d, 1H, 6-H); 7.92(d, 1H, 9-H).

**8-Chloro-1-*iso*-butyl-imidazo[4,5-c]quinolin-4-amine** **(4b).** ^1^H-NMR (DMSO_d6_) δ 1.02(d, 6H, *J*=6.5 Hz, 2CH_3_), 2.35(non, 1H, CH), 4.30(d, 2H, CH_2_),. 1.02(d, 6H, 2CH_3_); 2,18(m, 1H); 4.40(d, 2H, CH_2_) 6.75(brs 2H, NH_2_); 7.40(dd, *J=*8.9, 2.4 Hz, 1H, 7-H); 7.60(d, 1H, 6-H); 7.89(d, 1H, 6-H); 8.18(s, 1H, 2-H). IR (KBr) ν 567, 640, 794, 1087 cm^-1^.

**1-*Iso-*butyl-8-methylimidazo[4,5-c]quinolin-4-amine** **hydrochloride** (**4c**). 1H-NMR δ (DMSO_d6_) 0.92(d, 6H, *J=* 6.5 Hz, 2CH_3_); 2.12(non, 1H, CH); 2.50(s, 3H, CH_3_); 4.42(d, 2H, CH_2_); 7.55(dd, *J* = 8.4, 1.3 Hz, 7-H); 7.75(d, 1H, 6-H); 7.93(brs, 1H, 9-H); 8.50(s, 1H, 2-H), 13.55(brs, 3H, ^+^NH_3_).

**1-*Iso*-butylimidazo[4,5-c][1,8]naphthyridin-4-amine** (**4d**) 1H-NMR (DMSO_d6_) δ 0.85(d, 6H, *J*= 6.5 Hz, 2CH_3_); 2.11(m, 1H, CH); 4.41(d, 2H, CH_2_); 7.02(brs, 2H, NH_2_); 7.25(dd, 1H, 8-H); 8.40(d, 1H, 9-H); 8.60(d, 1H, 7-H). ν(KBr) 628; 671; 1377; 1562; 1639; 2360; 3174; 3286; 3649; 3672; 3749; 3799 cm^-1^.

**5-Amino-1-methyl-pyrazol-4-carbonitrile** (**5**). Ethoxymethylene aminonitrile (12.2 g, 10 mmol) was added to a cold (0 °C) solution of methylhydrazine (5.25 mL, 10 mmol) and NEt_3_ (8 mL, 60 mmol) in 100 mL THF. The mixture was heated overnight at 60°C. After cooling the mixture was concentrated under vacuum and the residue was triturated in AcOEt. Yield 55%. Mp 110 °C. ^1^H-NMR (CDCl_3_) δ 3.51(s, 3H, CH_3_); 6.54(brs, 2H, NH_2_) 7.50(s, 1H, pyrazol), ^13^C-NMR δ 34.94; 34.97; 72.70; 115.82; 140.24; 151.94.

**5-Iodo-1-methyl-imidazole-4-carbonitrile** (**6**)**.** The iodation procedure was performed as described for the synthesis of **2**. ^1^H-NMR (CDCl_3_) δ 3.93(s, 3H, CH_3_); 7.75(s, 1H, pyrazol). ^13^C-NMR δ 40.74(CH_3_); 90.28(C); 102.43(C); 13.42(C); 143.71(CH).

**5-(2-Aminophenyl)-1-methyl-pyrazole-4-carbonitrile** (**7a**)**.** The coupling of iodopyrazole with 2-aminophenylboronate was achieved as previously achieved for **3a**. ^1^H-NMR (CDCl_3_) δ 3.64(brs, 2H, NH_2_); 3.78(s, 3H, CH_3_); 6.80(d, *J*= 7.6 Hz, 1H, Aro); 6.87(t, 1H, Aro); 7.10(d, 1H, Aro); 7.32(t, 1H, Aro); 7.87(s, 1H, pyrazol).

**5-(2-Amino-5-chloro-phenyl)-1-methyl-pyrazole-4-carbonitrile** (**7b**)**.** ^1^H-NMR (CDCl_3_) δ 3.69(brs, 2H, NH_2_); 3.76(s, 3H, CH_3_); 6.76(d, *J=* 7,0 Hz, 1H, Aro); 7.11(s, 1H, Aro); 7.28(d, 1H, Aro); 7.87(s, 1H, pyrazol).

**Preparation of pyrazolo[4,3-c]quinolin-4-amines.** The cyclization of compounds **7** into the tricyclic heterocycles **8** was accomplished using the same conditions as for **4**.

**1-Methyl-pyrazolo[4,3-c]quinolin-4-amine hydrochloride** (**8a**)**.** ^1^H-NMR (400 MHz, DMSO_d6_) δ 4.42(s, 3H, CH_3_); 7.58(t, *J* = 7.5 Hz, 8-H ); 7.78(t, 1H, 7-H); 7.80(d, 1H, 6-H); 8.38(d, *J*= 7.5 Hz, 1H, 9-H); 8.67(s, 1H, 3-H); 8.60, 9.80, 13.80 (3 brs, 3H, NH_3_^+^).

**8-Chloro-1-methyl-pyrazolo[4,3-c]quinolin-4-amine** **hydrochloride** (**8b**)**.** ^1^H-NMR (DMSO_d6_) δ 4.46(s, 3H, CH_3_); 7.83(m, 2H, 6-H and 7-H); 8.35(s, 1H, 9-H); 8.70(s, 1H, pyrazol); 7.20, 8.80, 9.80(3 brs, 3H, NH_3_^+^) δ 37.79(CH_3_); 93.25(C); 113.22(C); 117.74(CH); 123.89(C); 130.13(CH); 131.93(CH); 142.06(CH) 143.19(C).
