# Supplementary figures and images for: The Toll-Like Receptor Agonist Imiquimod Is Active against Prions
Source: PLoS One. 2013 Aug 16;8(8):e72112. doi: 10.1371/journal.pone.0072112 (PMC3745460; doi:10.1371/journal.pone.0072112)

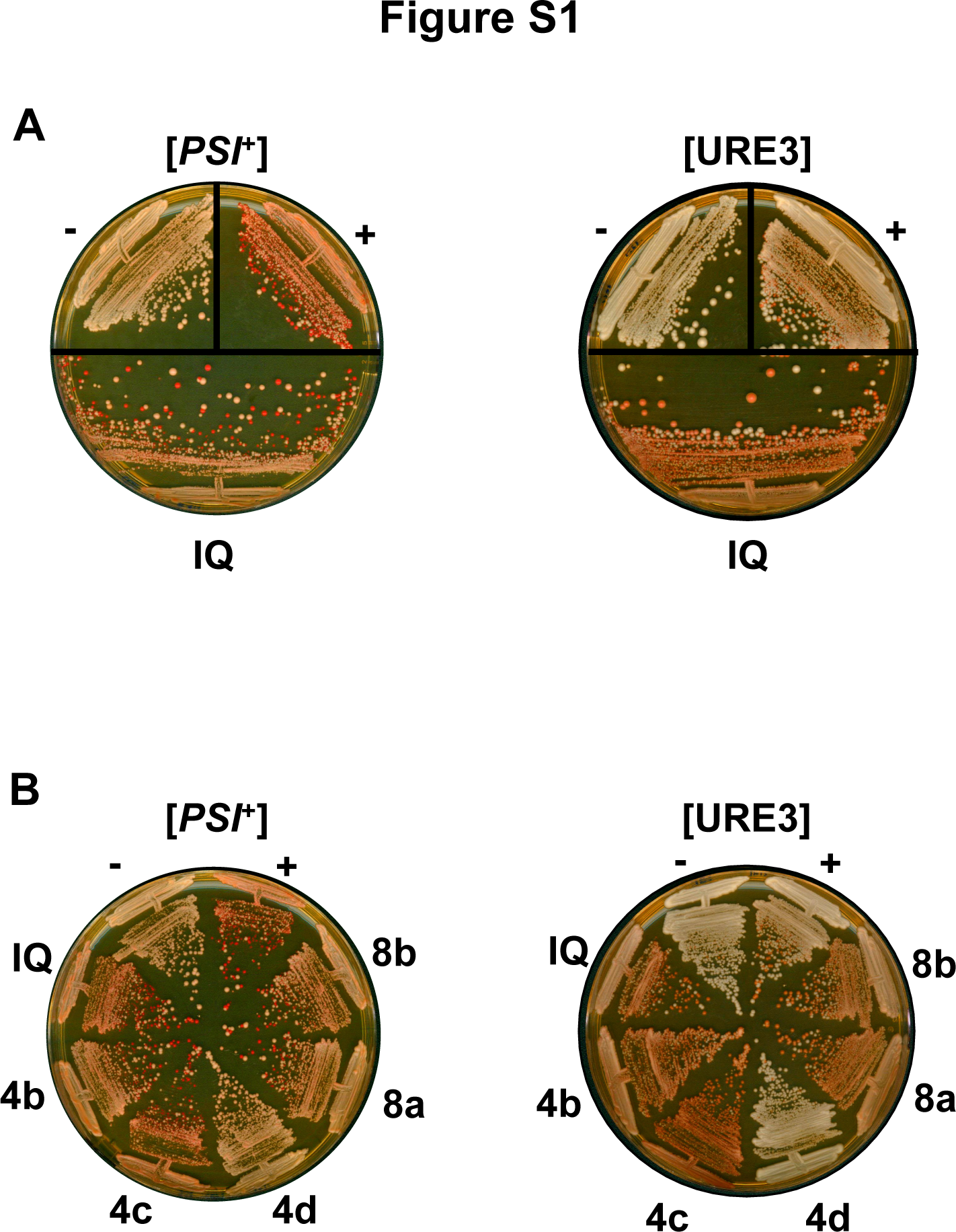

Supplement: Figure S1 — Cells from red halos surrounding filters on which IQ (A) or IQ, 4b, 4c, 4d, 8a, 8b (B) was loaded were streaked on drug-free YPD medium. Cells surrounding filters on which DMSO and GuHCl were loaded were used as negative and positive controls, respectively. (TIF) [file pone.0072112.s002.tif]
